# Emerging Clinical Role of Tavapadon, a Novel Dopamine Partial Agonist, in the Treatment of Parkinson’s Disease

**DOI:** 10.3390/diseases13090290

**Published:** 2025-09-02

**Authors:** Alan D. Kaye, Bennett M. Ford, Brennan M. Abbott, Kalob M. Broocks, Sofia Novacic, Sahar Shekoohi

**Affiliations:** 1 Department of Anesthesiology, Louisiana State University Health Sciences Center at Shreveport, Shreveport, LA 71103, USA; 2 School of Medicine, Louisiana State University Health Sciences Center, New Orleans, LA 70112, USA; bford8@lsuhsc.edu; 3 School of Medicine, Louisiana State University Health Sciences Center at Shreveport, Shreveport, LA 71103, USA

**Keywords:** Parkinson’s disease, Tavapadon, dopamine agonist, D1 receptor, clinical trials, motor symptoms, pharmacology

## Abstract

Tavapadon, a novel oral dopamine-D1R/D5R partial agonist, has been studied in recent years for the treatment of late-stage development Parkinson’s disease (PD). Levodopa, a dopamine precursor that currently remains the gold-standard first-line therapy for PD motor symptoms, serves as a benchmark against emerging dopaminergic agents. By selectively activating D1-family receptors on direct-pathway medium neurons, Tavapadon differs in that it delivers levodopa-level motor benefit while avoiding its many D2R/D3R-mediated adverse effects. In placebo-controlled trials, Tavapadon produced clear, clinically meaningful gains in motor function and day-to-day activities, as captured by the Unified Parkinson’s Disease Rating Scale (UPDRS). Recent late-stage results have revealed that Tavapadon maintains superior UPDRS outcomes in de novo patients and, when added to levodopa, extended “ON” time periods of reliable motor control free of troublesome dyskinesia, without introducing new safety concerns. In studies, nausea, headache, and somnolence were the most frequent adverse events. Hallucinations, orthostatic hypotension, and impulse-control disorders remained comparable to placebo, reflecting minimal D2R/D3R-mediated effects. Preclinical primate studies have demonstrated levodopa-like motor rescue with markedly less dyskinesia, a pattern mirrored in clinical add-on trials. Collectively, evidence indicates that Tavapadon can match levodopa-mediated symptomatic efficacy, lower dyskinesia liability compared with levodopa or earlier full D1 receptor (D1R) agonists, and offer the convenience of once-daily dosing characteristics, which may bridge the therapeutic gap between levodopa and the current D2R/D3R agonists in PD management. In the present investigation, the emerging clinical role for Tavapadon is described, along with the mechanism of action, clinical efficacy, safety, and future directions.

## 1. Introduction

Parkinson’s disease is a progressive neurodegenerative disorder that affects more than 10 million people worldwide [1] and results from a progressive loss of nigrostriatal dopaminergic neurons that govern motor function [2]. Levodopa, a dopamine precursor able to cross the blood–brain barrier, remains the therapeutic cornerstone for treatment because it provides the greatest symptomatic benefit for Parkinson’s disease [3,4]. In comparison to dopamine agonists, levodopa provides superior motor benefits in its treatment of Parkinson’s disease [5]. However, levodopa has been associated with major dopaminergic motor complications, mainly dyskinesia [4]. Levodopa-induced dyskinesia (LID) is due to rapid bursts of stimulation to dopamine receptors, the serotonin–neuron conversion of levodopa to dopamine, an overactive corticostriatal glutamate pathway, and overall abnormal activity on dopamine-releasing receptors [6]. Levodopa-induced dyskinesia (LID) appears in over 85% of patients after 9 to 15 years [4].

Dopamine targets two different receptors within the brain to coordinate movement. Primarily, the D1/D5 receptors (stimulate Gs and Golf proteins) act on the “direct” pathway and excite movement, and the D2/D3 receptors (stimulate Go and Gi proteins) act on the “indirect” pathway and inhibit excess movement [7]. D1 receptors are most commonly found in the basal ganglia and limbic motor areas such as the striatum, the nucleus accumbens, the substantia nigra, the olfactory bulb, and the cerebral cortex [8]. D2 receptors are much broader in their distribution, such as in the striatum, the nucleus accumbens, and the olfactory tubercle, but they are also present throughout the hippocampus, the amygdala, the hypothalamus, and other cortical areas [8]. Dopamine agonists thus restore damage to dopaminergic neurons lost to Parkinson’s disease by increasing dopamine activity in the brain [9]. The majority of dopamine agonists are D2 receptor agonists due to their preferential selection [9]. Common dopamine agonists such as Pramipexole, Ropinirole, and Rotigotine delay Levodopa initiation and reduce early dyskinesia risk [5], but their D2/D3 selectivity yields only moderate efficacy [2] and introduces limiting side effects such as hallucinations, nausea, orthostatic hypotension, edema, increased sleepiness, and impulse-control disorders [5].

These shortcomings have steered researchers towards a different strategy to selectively target the D1R pathway and match Levodopa motor efficacy by targeting the “direct” pathway while avoiding its long-term complications [10]. The “direct” (D1-driven) and “indirect” (D2-driven) model predicts that augmenting the direct pathway should restore movement with fewer complications because D2/D3 receptors are broader in their distribution, resulting in side effects such as those listed above [2,11]. First-generation full D1R agonists (ABT-431) validated this concept but were limited due to poor oral bioavailability, a short half-life, and severe peak-dose dyskinesias [10]. Tavapadon (CVL-751) was engineered to overcome these obstacles. Its non-catechol scaffold resists COMT/MAO metabolism, involving the two major enzymes that break down catecholamine molecules such as Levodopa [2]. It binds D1R and D5R with nanomolar potency while showing negligible activity at D2R–D4R, and it functions as a partial agonist, providing sub-maximal receptor activation [2].

## 2. Mechanism of Action and Pharmacology

Dopamine loss from Parkinson’s disease suppresses D1 receptor activation [8]; restoring it can, therefore, yield robust symptomatic improvement even when there is extreme nigrostriatal degeneration from Parkinson’s disease [12]. The D1 receptor is a G-protein-coupled receptor that activates adenylate cyclase, increasing cyclic AMP levels [8]. D1R activation depolarizes direct-pathway medium spiny neurons, facilitating thalamocortical feedback and voluntary movement [13]. Selective dopamine-like receptor full agonists that are catechol-derived activate D1R via interactions with transmembrane domain 5 (TM5) and transmembrane domain 3 (TM3) of the D1 receptor [14]. However, the constant activation of this receptor leads to the phosphorylation of the intracellular domains of the G-protein, causing eventual desensitization due to B-arrestin-mediated receptor endocytosis [14]. Tavapadon is a non-catechol agonist, and as such, it does not cause desensitization due to decreased recruitment of B-arrestin [14]. Tavapadon lacks interactions between TM5 and TM3 and activates D1R differently in that it interacts with the ECL2 domain of the receptor [14]. This suggests that Tavapadon differs in its ability to maintain D1 receptor activation without causing desensitization [14]. Further clarifying the mechanism through which Tavapdon limits B-arrestin endocytosis, a structural study investigating Tavapdon’s binding mechanism demonstrated that certain ligands exhibit their own specific bias agonism to the D1R that can favor G-protein or B-arrestin signaling, depending on their positioning within the receptor [15]. Specifically, the interactions of ligands within the TM5 domain are crucial, as the relative distance between the ligand and residue S198^5.42^ determines the confirmational flexibility needed for efficient B-arrestin coupling [15]. Tavapadon has an extra oxygen atom, thus positioning it closer to residue S198^5.42^ within TM5, and it sterically restricts this flexibility, thus reducing B-arrestin endocytosis [15]. As a result, Tavapdon favors G-protein singling without causing desensitization via B-arrestin-mediated endocytosis.

Tavapadon shows a sub-nanomolar higher affinity for D1R (*K*_i_ = 9 nM) and D5R (*K*_i_ = 13 nM) receptors and a low affinity for D2R (*K*_i_ ≥ 6210 nM), D3R (*K*_i_ ≥ 6720 nM), and D4R (*K*_i_ ≥ 4870 nM) [2]. Functional assays reveal Tavapadon acting as a partial agonist, with 65% of dopamine’s intrinsic ability for D1R and 81% for D5R [2]. Acting as a partial agonist, Tavapadon still supplies enough stimulation for motor benefit, yet it can still blunt receptor over-activation, limiting dyskinesia, a concept validated in non-human primates [16].

D2R/D3R agonists modulate the indirect pathway and numerous extra striatal circuits due to their broader distribution [8]. This indirect pathway activation causes adverse side effects such as somnolence, orthostatic hypotension, hallucinations, and impulse-control disorders [5]. Tavapadon bypasses these liabilities by sparing D2R/D3R [2]. In contrast to these dopamine agonists, levodopa floods all receptors, delivering unmatched potency but triggering pulsatile surges that drive LID [6]. Tavapadon provides a steadier direct-pathway stimulus, attenuating fluctuation amplitude and, when used adjunctively, allowing a levodopa dose reduction that lessens the dyskinesia risk [6,17].

## 3. Clinical Trial Evidence

Clinical trials have assessed the efficacy of Tavapadon in treating various stages of Parkinson’s disease. These trials enrolled diverse patient populations, from those newly diagnosed to those with advanced motor symptoms of PD. The purpose of these trials ranges from establishing the pharmacologic profile of Tavapadon in PD to the use of Tavapadon as an adjunct to mainstay treatment. This section summarizes key clinical trials, highlighting patient characteristics, outcome measures, and efficacy findings supporting Tavapadon-mediated therapeutic potential in PD management.

An early clinical trial by Sohur et al. was designed to establish the pharmacokinetic and pharmacodynamic profile of Tavapadon in PD patients. This Phase I study incorporated a single ascending dose (SAD) cohort and multiple ascending dose (MAD) cohort [18]. The SAD study utilized a double-blind, three-way crossover design assessing 18 patients with PD. Patients were administered ascending doses of Tavapadon or placebo once during the treatment period for three treatment periods with a seven-day drug washout between treatments. The doses of Tavapadon used were 0.75, 1.5, 3, 6, and 9 mg [18]. The MAD study utilized an open-label design including 45 patients with PD. Patients were administered Tavapadon once daily for 21 consecutive days, titrating up to either 5, 15, or 25 mg once daily. Modern PD trials rely on the Movement Disorder Society-Unified Parkinson’s Disease Rating Scale (MDS-UPDRS) [19,20]. Part III quantifies motor signs, while Part II gauges activities of daily living [21]. The primary outcome of both studies was the change in MDS-UPDRS III score, either from the baseline or at different times post-dose [18]. In the SAD study, Tavapadon at a dose of 9 mg significantly reduced MDS-UPDRS-III scores vs. placebo at 12 h post-dose (−11.13 ± 3.68, *p* = 0.0028), while 3 mg showed a non-significant decrease. In the MAD study, Tavapadon at 15 mg and 25 mg significantly decreased the MDS-UPDRS III score at 12 h post-dose (–20.0 ± 12.90 and –9.33 ± 14.60, respectively), while 5 mg led to an insignificant increase [18]. Overall, Tavapadon was reported to exert sustained pharmacodynamic effects in the treatment of PD patients, supporting larger clinical trials on its therapeutic potential [18].

Adding to these early findings, a Phase II double-blind, placebo-controlled trial by Riesenberg et al. assessed the efficacy of Tavapadon as monotherapy for patients with early-stage PD. Fifty-seven patients with early-stage PD, having not been treated with dopaminergic agonists for any longer than 28 days, were randomized to receive flexible dosing from 0.25 to 15 mg of daily Tavapadon over 15 weeks. The primary measure of efficacy was the change from baseline MDS-UPDRS III scores. The Tavapadon treatment groups experienced a significant mean decrease in MDS-UPDRS III vs. placebo at week 15 of 4.8 (*p* = 0.0407). The study also noted significant improvements in MDS-UPDRS III scores at all time points prior to week 15. It was concluded that Tavapadon improves motor function and is well tolerated in patients with early-stage PD [19]. These results are encouraging; however, it should be noted that the overall size of the patient population was small in both Phase I (n = 18) and II (n = 57), limiting the strength of evidence. Phase III TEMPO 1–3, described below, included a much larger cohort, and it provides more robust support for Tavapdon’s efficacy in treating PD. While TEMPO 4 trials have enrolled even larger cohorts, these current results of Phases 1 and 2 are more limited and should be interpreted with more caution, given their sample size.

The TEMPO clinical trial program further expanded the evaluation of Tavapadon in the treatment of PD through four Phase III studies. The TEMPO-1 and 2 studies evaluated Tavapadon effectiveness as a monotherapy, utilizing fixed dose or flexible dosing, respectively. Combined MDS-UPDRS II and III scores were the primary endpoint in both studies. TEMPO-1 assigned 529 patients with PD randomly to either placebo, 5 or 15 mg of Tavapadon once daily for 27 weeks. Patients treated with 5 mg and 15 mg had significantly reduced MDS-UPDRS scores compared with placebo at the end of the study period (−9.7, *p* < 0.0001 and −10.2, *p* < 0.0001, respectively). TEMPO-2, on the other hand, enrolled 304 patients with PD and randomly assigned them 1:1 to either placebo or Tavapadon 5 mg to 15 mg titrated to the maximum tolerated dose for 27 weeks. Tavapadon-treated patients had a significantly reduced MDS-UPDRS II and III combined score vs. placebo at the end of the study period of −9.1, *p* < 0.0001 [22,23,24]. These Phase III trials provided further evidence supporting Tavapadon efficacy in motor symptom control for PD.

The TEMPO-3 Phase III clinical trial followed up on the earlier TEMPO trials by evaluating Tavapadon efficacy as an adjunctive treatment to levodopa therapy for motor symptoms of PD. Five hundred and seven patients who were experiencing motor fluctuations despite being on a stable dose of levodopa for at least 4 weeks were enrolled in the trial. Patients were randomly assigned to either a placebo or flexible-dose Tavapadon at 5–15 mg once daily for 27 weeks. The primary endpoint was the change from the baseline in “on” time without troublesome dyskinesia. The “on” time refers to the time period when a patient has good motor control of their symptoms. Patients treated with Tavapadon as an adjunct to levodopa experienced a significant increase in their total “on” time without troublesome dyskinesia vs. those treated with levodopa and an adjunct placebo (17 h vs. 0.6 h, *p* < 0.0001) [25]. These findings elucidated the ability of Tavapadon to manage motor fluctuations when combined with levodopa, which may be especially beneficial as PD advances.

TEMPO-4 is an ongoing trial that aims to assess the long-term effectiveness of Tavapadon’s management of motor symptoms. This open-label trial recruited 992 patients from previous TEMPO trials and administered 5–15 mg of Tavapadon once daily over 58 weeks. The final data for this trial is expected in January 2026 and will provide insights into the long-term efficacy and safety of Tavapadon for PD treatment. Importantly, this trial aims to measure adverse side effects to Tavapadon, such as clinically significant abnormalities in clinical laboratory evaluations, vital signs, physical and neural evaluations, and ECGs [26]. These results will hopefully provide more insight into longer-term side effects, as current studies are again more limited in their evaluations due to smaller sample sizes. Table 1 summarizes key findings from different therapies for PD.

## 4. Safety and Tolerability

Early preclinical and clinical studies of the D1 receptor agonist showed proof of having anti-Parkinsonian motor effects in patients both with and without severe dyskinesias [18]. However, these effects were mitigated by the fact that these drugs had poor bioavailability, a short half-life, and low tolerability, thus limiting their clinical use [10]. The safety profiles for Tavapadon have thus outdated this earlier research by having many of the identified adverse events described only as mild to moderate in severity, such as headaches, nausea, or vomiting, an increased half-life, and increased bioavailability, reflecting its selectivity as a D1R/D5R partial agonist [18,19,29].

The Phase I trial conducted by Sohur et al. evaluated Tavapadon in both single-ascending-dose (SAD) and multiple-ascending-dose (MAD) studies [18]. The SAD study showed that single doses of 9 mg were safe and tolerable in Parkinson’s patients without any deaths, severe adverse events, discontinuations, or dose reductions [18]. Any adverse events that were monitored were mild to moderate in severity and mostly limited to headache, nausea, and vomiting [18]. The MAD study reported similar side effects, with the exception of six severe adverse events; however, all of these effects occurred during the up-titration of the drug [18]. Also, these incidences seem to be related to the increment and pace of the up-titration, rather than exposure to the drug [18].

Along with its minimal side effects, Tavapadon was also shown to be generally well tolerated on the neuropsychiatric scale. It showed no significant differences in depression, sleep, suicide severity, or behavioral changes. These changes were compared and scored on the Beck Depression Inventory-II scale, Epworth Sleepiness scale, the Columbia Suicide Severity Rating scale, and the Questionnaire for Impulsive Compulsive Disorders in Parkinson’s Disease scale, respectively [19]. Tavapadon has been shown so far to present a better side effect profile than both placebo and other concurrent Parkinson’s drugs [4,18]. Levodopa is known to cause nausea, dyskinesia, and hallucinations, and over time, it is associated with fluctuations in efficacy and a decrease in duration, leading to up-titrated doses [4,27]. Along with these effects, up to 40% of patients taking levodopa experience motor fluctuations 3–5 years after starting therapy [18] While studies involving Tavapadon have not assessed long-term effects, there has been no evidence showing that this drug might be associated with these “ON/OFF” fluctuations. More studies need to be conducted to determine long-term tolerability.

D2R/D3R agonists, such as Pramipexole and Ropinirole, are associated with edema, nausea, hallucinations, increased sleepiness, and impulse-control disorders [4]. So far, there has been no evidence to support the notion that D1R/D5R dopamine receptors, which deal with the direct pathway, can cause impulse-control disorders like the drugs listed above [1]. However, the majority of these studies have been more limited to drug-seeking behavior research [1]. Most adverse events are mild to moderate in severity and include nausea, headache, dry mouth, and tremor [5,18,19]. There was noted to be a decrease in systolic and diastolic blood pressure, but these changes were non-significant, and cardiovascular function was unaffected [4,18,19]. Overall, Tavapadon exhibits a mild side effect profile and good tolerability in early-stage Parkinson’s patients, as shown in Table 2.

## 5. Patient Outcomes and Dosing Convenience

Multiple studies have demonstrated Tavapadon to be safe and effective in treating Parkinson’s disease (PD) [18,19]. The half-life of Tavapadon is approximately 24 h, and thus, its ability to be dosed once daily adds to its appeal by potentially improving patient adherence [29]. Tavapadon has a much longer half-life than levodopa; thus, patients are more prone to an “on-off” effect through which patients experience periods of symptom control, followed by a return of symptoms, when using only Levopoda [27]. This requires patients on levodopa therapy to dose multiple times a day as well [27]. The relatively long half-life of Tavapadon in comparison makes it more convenient to take and reduces the motor fluctuations associated with shorter-acting medications such as levodopa [27,38]. This may improve patient adherence and overall patient quality of life. Tavapadon’s pharmacologic properties and clinical outcomes make it a promising alternative to traditional PD treatments.

The different metabolic route of Tavapdon influences clinical outcomes as well. Tavapdon is cleared via cytochrome P450 (CYP450) 3A4 (CYP3A4) with a minimal renal excretion of <2% [29]. Of note, high-fat meals did not affect the rate of absorption [29]. In comparison, levodopa is susceptible to quick peripheral metabolism via amino acid decarboxylase, COMT, and MAO enzymes, which leads to the fluctuating dopamine levels associated with dyskinesia [27]. Thus, the ability of Tavapadon to be unaffected by peripheral metabolism further supports smoother symptom control and fewer complications compared to Levopoda.

## 6. Future Directions and Emerging Research

The role of Tavapadon as an adjunctive treatment has also been studied. The TEMPO-3 trial showed once-daily Tavapadon was an effective adjunctive therapy to levodopa for the treatment of PD. Patients who used levodopa for at least 4 weeks prior to the study received either 5–15 mg of Tavapadon or a placebo. Patients who received Tavapadon therapy in addition to their levodopa had a significant increase of 1.1 h in the daily “on” time without troublesome dyskinesia compared to placebo patients only receiving levodopa. Patients receiving the combination therapy also had a significant reduction in daily “off” time of 0.94 h less daily. Most adverse effects were reported as mild or moderate [25]. Combination therapy is especially important in patients with advanced stages of PD, when motor symptoms become progressively harder to control. The ability of Tavapadon to selectively target the D1/D5 dopamine receptor offers a new pathway for treatment compared to existing treatments such as levodopa and traditional dopamine agonists, which primarily target the D2/D3 receptor [2]. The complementary mechanisms may allow for more flexible treatment instead of relying on a single dopaminergic pathway.

Currently underway, TEMPO-4 is an open-label Phase III clinical trial being conducted to evaluate the safety and efficacy of long-term Tavapadon therapy in patients with PD. Patients will take once-daily Tavapadon therapy at dosages of 5–15 mg for a duration of 58 weeks to determine long-term outcomes of therapy [24,37]. This study will also offer helpful insights into patient adherence. This clinical trial is estimated to be completed in January 2026. AbbVie Inc., the pharmaceutical company leading the development of Tavapadon, plans to submit a New Drug Application (NDA) to the U.S. Food and Drug Administration (FDA) in 2025 [24]. This would make Tavapadon the first selective D1/D5 dopamine receptor agonist available for PD treatment if approved.

## 7. Conclusions

Parkinson’s disease (PD) is a progressive neuro-degenerative disorder caused by the selective degeneration of nigrostriatal dopamine neurons, culminating in steadily worsening motor disability [4]. Levodopa remains the benchmark therapy, yet its ~90 min plasma half-life and pulsatile receptor stimulation drive wearing-off and Levodopa-induced dyskinesia (LID) in up to 80% of patients after a decade [6,27,38]. D2/D3-selective agonists can defer levodopa but provide only moderate efficacy and are hampered by somnolence, hallucinations, orthostatic hypotension, and impulse-control disorders [5].

Tavapadon (CVL-751) was engineered to overcome these shortcomings. Its non-catechol structure is able to resist COMT/MAO peripheral metabolism, giving an elimination half-life of ~24 h and enabling true once-daily dosing [2,29]. The compound exhibits a nanomolar affinity and partial-agonist efficacy at D1R/D5R receptors with much greater fold selectivity over D2R-like subtypes [2,14]. In Parkinsonian macaques, sustained D1R stimulation restored locomotion while eliciting markedly less dyskinesia than levodopa [16], supporting the concept that continuous direct-pathway activation can deliver robust benefit without sensitizing motor circuits.

A comprehensive Phase III program is now underway. TEMPO-1 and TEMPO-2 assess fixed- and flexible-dose Tavapadon over 27 weeks in drug-naïve patients, while TEMPO-3 evaluates Tavapadon as an adjunct to levodopa [20]. At the 2025 American Academy of Neurology meeting, Fernandez et al. (2025) reported that adjunctive Tavapadon lengthened daily “good ON” time by ~1.1 h and cut “OFF” periods by nearly 1 h without increasing troublesome dyskinesia, aligning with the promise of continuous D1R/D5R activation [20]. Feasibility work in advanced PD further indicates that partial D1R agonism retains efficacy when conventional options fail [11]. Disease-progression modeling predicts that introducing selective D1R stimulation at diagnosis could delay Levodopa initiation by one to two years and halve the cumulative dyskinesia risk [13].

By combining pharmacokinetic stability with high D1R/D5R selectivity, Tavapadon offers a rational strategy for restoring physiological basal-ganglia output while minimizing the liabilities of existing dopaminergic drugs. Randomized data already demonstrate clinically meaningful motor improvements and extension of “good ON” time with a benign neuro-psychiatric profile. Long-term safety, durability, and non-motor outcomes are being clarified through the 58-week TEMPO-4 extension. Should these studies confirm initial findings, Tavapadon is poised to expand the therapeutic armamentarium for PD, allowing an earlier, sustained engagement of the direct dopaminergic pathway and potentially redefining guideline algorithms across the disease continuum.

## Figures and Tables

**Table 1:** Comparison of Tavapadon with standard Parkinson’s disease therapies.

Parameter	Tavapadon	Levodopa	D2/D3 Agonists (e.g., Pramipexole)
Mechanism of Action	Selective partial D1/D5 dopamine agonist [2]	Non-selective dopamine precursor [27]	D2/D3 full agonist [2]
Formulations	Oral release tablet [18]	Immediate/controlled-release tablets, controlled-release capsules, Enteral infusion, Inhalation powder [28]	Oral tablet, transdermal patch, subcutaneous injection [9]
Metabolism Pathway	Primarily CYP3A4 [29]	MAO-B and COMT [30]	Various hepatic pathways [31]
Half-life	~24 h [29]	~60–90 min [27]	5–12 h [9]
Dosing frequency	Once daily [29]	2–6 times daily [27]	1–3 times daily [2]
FDA approval status	Not FDA-approved [32]	FDA-approved PD treatment [32]	FDA-approved PD treatment [32]
Common Side effects	Nausea, headache, dizziness [33]	Dyskinesias, severe on-off motor fluctuations [34]	Impulsive behavior problems (e.g., gambling, hypersexual) [34]

**Table 2:** Clinical efficacy and safety of Tavapadon in Parkinson’s disease.

Author (Year)	Phase	Intervention	Results	Conclusion
Sohur et al. (2018) [18]	Phase I	Single and multiple ascending doses of Tavapadon 0.25–25 mg once or multiple doses daily for 21 days	MDS-UPDRS-III significantly decreased (−11.13 ± 3.68) in the 9-mg treatment group vs. placebo.	Tavapadon was efficacious, safe, and well tolerated. These findings warrant further clinical trials.
Risenberg et al. (2020) [19]	Phase II	Flexible-dose Tavapadon once daily for 15 weeks	MDS-UPDRS-III scores in Tavapadon-treated patients significantly improved by 4.8 ± 2.26 points compared to placebo at 15 weeks. Significant improvements in MDS-UPDRS-III were also seen at all assessment points before 15 weeks.	Tavapadon once daily resulted in significant improvement of motor symptoms and was generally well tolerated.
TEMPO-1 (NCT04201093), Cerevel Therapeutics (2024) [22]	Phase III	Fixed dose of Tavapadon 5 or 15 mg once daily for 27 weeks	MDS-UPDRS-III scores in both 5 mg and 15 mg groups significantly improved compared to placebo at 26 weeks. Placebo: +1.8 5 mg: −9.7; 15 mg: −10.2; *p*-value < 0.0001 versus placebo	Tavapadon demonstrated significant and clinically meaningful improvement in motor symptoms in PD patients.
TEMPO-2 (NCT04223193), AbbVie (2024) [24,35]	Phase III	Flexible dose of Tavapadon 5–15 mg once daily for 27 weeks	MDS-UPDRS-II and III significantly improved compared to placebo at 26 weeks. Placebo: −1.2 5–15 mg: −10.3; *p*-value < 0.0001 versus placebo.	Tavapadon demonstrated significant and clinically meaningful improvement in motor symptoms in PD patients.
TEMPO-3 (NCT04542499), AbbVie (2024) [36]	Phase III	Tavapadon flexible dose for 27 weeks as an adjunct to levodopa therapy	“On” and “Off” times significantly improved in Tavapadon-adjunctive-treated patients compared with levodopa-only patients. +1.1 h “On” time without dyskinesia (1.7 h vs. 0.6 h; *p*-value < 0.0001) - Statistically significant “off” time observed in Tavapadon treatment arm.	Tavapadon is effective as adjunctive therapy in advanced PD with motor fluctuations.
TEMPO-4 (NCT04760769), AbbVie (estimated 2026) [37]	Phase III	Tavapadon 5–15 mg once daily for 58 weeks	Ongoing. Assessing long-term safety, adherence, and control of motor symptoms.	Ongoing. Will provide information concerning the long-term efficacy and tolerability of Tavapadon therapy for PD.

## Data Availability

Data sharing is not applicable to this article, as no datasets were generated or analyzed during the current study.
